# 2-(5,6-Dihydro­benzimidazo[1,2-*c*]quinazolin-6-yl)phenol

**DOI:** 10.1107/S1600536811030583

**Published:** 2011-08-06

**Authors:** Naser Eltaher Eltayeb, Siang Guan Teoh, Ching Kheng Quah, Hoong-Kun Fun

**Affiliations:** a Department of Chemistry, Faculty of Pure and Applied Sciences, International University of Africa, Sudan, and, School of Chemical Sciences, Universiti Sains Malaysia, 11800 USM, Penang, Malaysia; b School of Chemical Sciences, Universiti Sains Malaysia, 11800 USM, Penang, Malaysia; c X-ray Crystallography Unit, School of Physics, Universiti Sains Malaysia, 11800 USM, Penang, Malaysia

## Abstract

The asymmetric unit of the title compound, C_20_H_15_N_3_O, contains two independent mol­ecules, each of which is disordered over two sets of sites corresponding to a rotation of approximately 180° of the dihydro­benzimidazoquinazoline moiety, with refined site occupancies of 0.7479 (13) and 0.2521 (12) for both mol­ecules. The pyrimidine rings are in sofa conformations. In one mol­ecule, the hy­droxy-substituted benzene ring forms dihedral angles of 83.9 (3) and 82.4 (4)° for the major and minor components, respectively, with the mean plane of the benzimidazole ring system. The corres­ponding dihedral angles in the other mol­ecule are 88.31 (14) and 85.8 (6)°. In the crystal, mol­ecules are linked *via* inter­molecular O—H⋯N and N—H.·O hydrogen bonds into chains along [100].

## Related literature

For general background to and the biological activity of benzimidazole compounds, see: Minoura *et al.* (2004 ▶); Pawar *et al.* (2004 ▶); Tomei *et al.* (2003 ▶); Barreca *et al.* (2003) ▶; Demirayak *et al.* (2002 ▶). For the synthesis, see: Eltayeb *et al.* (2011 ▶). For related structures, see: Eltayeb *et al.* (2007*a* ▶,*b* ▶,*c* ▶, 2009 ▶). For bond-length data, see: Allen *et al.* (1987 ▶). For ring conformations, see: Cremer & Pople (1975 ▶).
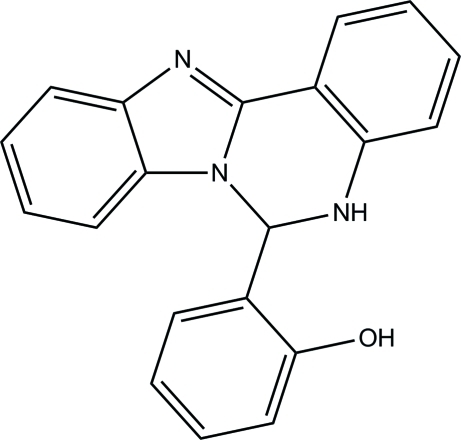


## Experimental

### 

#### Crystal data

C_20_H_15_N_3_O
*M* *_r_* = 313.35
Monoclinic, 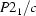

*a* = 17.1513 (10) Å
*b* = 19.1160 (11) Å
*c* = 9.2630 (5) Å
β = 97.590 (1)°
*V* = 3010.4 (3) Å^3^
*Z* = 8
Mo *K*α radiation
μ = 0.09 mm^−1^
*T* = 297 K
0.30 × 0.28 × 0.25 mm

#### Data collection

Bruker APEXII DUO CCD area-detector diffractometer
Absorption correction: multi-scan (*SADABS*; Bruker, 2009 ▶) *T*_min_ = 0.974, *T*_max_ = 0.978
40861 measured reflections
11057 independent reflections
8231 reflections with *I* > 2σ(*I*)
*R*_int_ = 0.047

#### Refinement

*R*[*F* ^2^ > 2σ(*F* ^2^)] = 0.058
*wR*(*F* ^2^) = 0.158
*S* = 1.05
11057 reflections
625 parameters
144 restraints
H-atom parameters constrained
Δρ_max_ = 0.44 e Å^−3^
Δρ_min_ = −0.42 e Å^−3^

### 

Data collection: *APEX2* (Bruker, 2009 ▶); cell refinement: *SAINT*; data reduction: *SAINT* (Bruker, 2009 ▶); program(s) used to solve structure: *SHELXTL* (Sheldrick, 2008 ▶); program(s) used to refine structure: *SHELXTL*; molecular graphics: *SHELXTL*; software used to prepare material for publication: *SHELXTL* and *PLATON* (Spek, 2009 ▶).

## Supplementary Material

Crystal structure: contains datablock(s) global, I. DOI: 10.1107/S1600536811030583/lh5291sup1.cif

Structure factors: contains datablock(s) I. DOI: 10.1107/S1600536811030583/lh5291Isup2.hkl

Supplementary material file. DOI: 10.1107/S1600536811030583/lh5291Isup3.cml

Additional supplementary materials:  crystallographic information; 3D view; checkCIF report

## Figures and Tables

**Table 1:** Hydrogen-bond geometry (Å, °)

*D*—H⋯*A*	*D*—H	H⋯*A*	*D*⋯*A*	*D*—H⋯*A*
O1*A*—H1*OA*⋯N1*B*^i^	0.98	1.69	2.641 (3)	162
N3*A*—H3*AB*⋯O1*A*^ii^	0.86	2.50	3.241 (3)	144
O1*B*—H1*OB*⋯N1*A*^iii^	0.97	1.79	2.686 (4)	151
N3*B*—H3*BB*⋯O1*B*^iv^	0.86	2.15	2.974 (4)	160

Symmetry codes: (i) 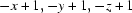; (ii) 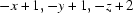; (iii) 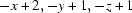; (iv) 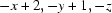.

**Fig. 1.**
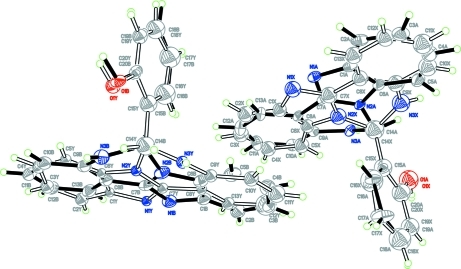
The molecular structure of the title compound showing 50% probability displacement ellipsoids for non-H atoms. All components are shown.

**Fig. 2.**
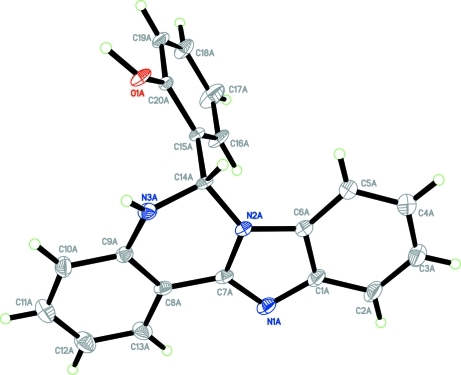
The molecular structure of the major component of molecule A showing 50% probability displacement ellipsoids for non-H atoms.

**Fig. 3.**
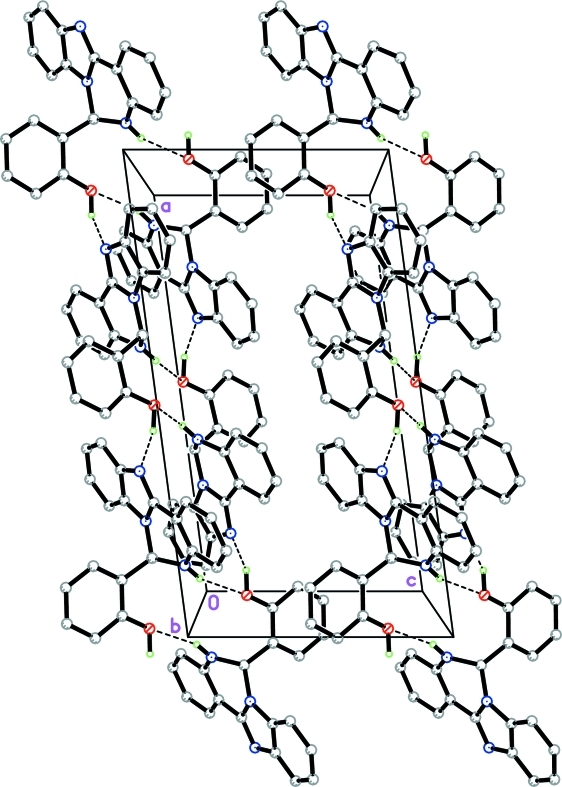
Part of the crystal structure of the title compound, viewed along the b axis. H atoms not involved in hydrogen bonds (dashed lines) have been omitted for clarity. Only the major disorder component is shown.

## supplementary crystallographic information

### Comment

The synthesis of benzimidazoles has received much attention owing to the varied biological activity such as antidiabetic (Minoura *et al.*, 2004), antimicrobial, antifungal (Pawar *et al.*, 2004), antiviral (Tomei *et al.*, 2003), antiHIV (Barreca *et al.*, 2003) and anticancer (Demirayak *et al.*, 2002) properties exhibited by a number of derivatives of these compounds. Previously we reported the crystal structures of  4-allyl-2-[1-(5-allyl-2-hydroxy-3-methoxybenzyl) -1H-benzimidazol-2-yl]-6-methoxyphenol (Eltayeb *et al.*, 2007*a*), 2-(2-methoxynaphthalen-1-yl)-1-[(2-methoxynaphthalen-1-yl)methyl] -1H-benzimidazole (Eltayeb *et al.*, 2007*b*), 2-(benzimidazol-2-yl) -6-methoxyphenol (Eltayeb *et al.*, 2007*c*) and 2-methoxy-6-(6-methyl -1H-benzimidazol-2-yl)phenol (Eltayeb *et al.*, 2009). Considering the biological importance of the included benzimidazole ring, we describe in this paper the single crystal X-ray diffraction study of 2-(5,6- dihydrobenzimidazo[1,2-c]quinazolin-6-yl)phenol.

The asymmetric unit contains two independent molecules (Fig. 1), *A* and *B*. In each molecule, the benzimidazole fused ring system is disordered over two sets of sites with refined site occupancies of 0.748 (1) : 0.252 (1), suggesting 180° rotational disorder for the benzimidazole fused ring system. The conformations for pyrimidine rings are close to a sofa conformation (Cremer & Pople, 1975), puckering parameters Q = 0.330 (2) Å, Θ =  61.9 (3)° and φ = 277.5 (4)° (major component of molecule *A*), Q =  0.305 (12) Å, Θ = 120.6 (19)° and φ = 103.9 (19)° (minor component of molecule *A*), Q =  0.343 (3) Å, Θ =  60.4 (4)° and φ = 285.8 (4)°  (major component of molecule *B*) and Q =  0.331 (11) Å, Θ =  119.3 (14)° and φ = 104.7 (14)° (minor component of molecule *B*). Bond lengths (Allen *et al.*, 1987) and angles are within normal ranges. In molecule *A* (Fig. 2), the hydroxyphenyl ring (C15-C20) is almost perpendicular to the mean plane of benzimidazole ring (N1/N2/C1-C7) with the dihedral angles of 83.9 (3) and 82.4 (4)° for major and minor components, respectively. The corresponding dihedral angles for molecule *B* are 88.31 (14) and 85.8 (6)°.

In the crystal structure, Fig. 3, the molecules are linked *via* intermolecular O1A–H10A···N1B^i^, N3A–H3AB···O1A^ii^, O1B–H10B···N1A^iii^ and N3B–H3BB..O1B^iv^ hydrogen bonds (Table 1) into infinite one-dimensional chains along [100].

### Experimental

The title compound was synthesized using Taha-Teoh's method (Eltayeb *et al.*, 2011). To a solution of 2-(2-aminophenyl)-1H-benzimidazole (0.209 g, 1.0 mmol) in ethanol (30 mL) was added 2-hydroxybenzaldehyde (0.1 mL, 1.0 mmol). The color of the resulting solution is pale-yellow. Then on adding zinc chloride (0.136 g, 1.0 mmol), the color of the solution became golden-yellow. The mixture was refluxed with stirring for 3 h. Crystals suitable for XRD were formed after several days of slow evaporation of ethanol at room temperature.

### Refinement

The title compound is disordered over two positions with refined site-occupancies of 0.748 (1) : 0.252 (1). All minor disordered components were refined isotropically. The O-bound hydrogen atoms were located from difference Fourier map and refined as riding on their parent atom, with *U*_iso_(H) = 1.2 *U*_eq_(O). The rest of the hydrogen atoms were positioned geomatrically [C–H = 0.93 - 0.98 Å; N–H = 0.86 Å] and refined using a riding model, with *U*_iso_(H) = 1.2 *U*_eq_(C, N). The highest residual electron density peak and the deepest hole are located at 0.68 Å and 0.12 Å, respectively, from H3AB. The same U^ij^ parameters were used for atom pair C16Y/C12Y.

### Crystal data

**Table:**

C_20_H_15_N_3_O	*F*(000) = 1312
*M**_r_* = 313.35	*D*_x_ = 1.383 Mg m^−^^3^
Monoclinic, *P*2_1_/*c*	Mo *K*α radiation, λ = 0.71073 Å
Hall symbol: -P 2ybc	Cell parameters from 9430 reflections
*a* = 17.1513 (10) Å	θ = 2.4–32.7°
*b* = 19.1160 (11) Å	µ = 0.09 mm^−^^1^
*c* = 9.2630 (5) Å	*T* = 297 K
β = 97.590 (1)°	Block, yellow
*V* = 3010.4 (3) Å^3^	0.30 × 0.28 × 0.25 mm
*Z* = 8	

### Data collection

**Table:**

Bruker APEXII DUO CCD area-detector diffractometer	11057 independent reflections
Radiation source: fine-focus sealed tube	8231 reflections with *I* > 2σ(*I*)
graphite	*R*_int_ = 0.047
φ and ω scans	θ_max_ = 32.8°, θ_min_ = 2.4°
Absorption correction: multi-scan (*SADABS*; Bruker, 2009)	*h* = −26→26
*T*_min_ = 0.974, *T*_max_ = 0.978	*k* = −29→29
40861 measured reflections	*l* = −12→14

### Refinement

**Table:**

Refinement on *F*^2^	Primary atom site location: structure-invariant direct methods
Least-squares matrix: full	Secondary atom site location: difference Fourier map
*R*[*F*^2^ > 2σ(*F*^2^)] = 0.058	Hydrogen site location: inferred from neighbouring sites
*wR*(*F*^2^) = 0.158	H-atom parameters constrained
*S* = 1.05	*w* = 1/[σ^2^(*F*_o_^2^) + (0.0604*P*)^2^ + 1.3534*P*] where *P* = (*F*_o_^2^ + 2*F*_c_^2^)/3
11057 reflections	(Δ/σ)_max_ = 0.001
625 parameters	Δρ_max_ = 0.44 e Å^−^^3^
144 restraints	Δρ_min_ = −0.42 e Å^−^^3^

### Special details

**Table:**

Geometry. All esds (except the esd in the dihedral angle between two l.s. planes) are estimated using the full covariance matrix. The cell esds are taken into account individually in the estimation of esds in distances, angles and torsion angles; correlations between esds in cell parameters are only used when they are defined by crystal symmetry. An approximate (isotropic) treatment of cell esds is used for estimating esds involving l.s. planes.
Refinement. Refinement of F^2^ against ALL reflections. The weighted R-factor wR and goodness of fit S are based on F^2^, conventional R-factors R are based on F, with F set to zero for negative F^2^. The threshold expression of F^2^ > 2sigma(F^2^) is used only for calculating R-factors(gt) etc. and is not relevant to the choice of reflections for refinement. R-factors based on F^2^ are statistically about twice as large as those based on F, and R- factors based on ALL data will be even larger.

### Fractional atomic coordinates and isotropic or equivalent isotropic displacement parameters (Å^2^)

**Table:**

	*x*	*y*	*z*	*U*_iso_*/*U*_eq_	Occ. (<1)
O1A	0.47310 (12)	0.61154 (16)	0.9445 (3)	0.0161 (5)	0.7479 (13)
H1OA	0.4159	0.6051	0.9253	0.019*	0.7479 (13)
N1A	0.82502 (7)	0.56968 (7)	0.82493 (16)	0.0190 (3)	0.7479 (13)
N2A	0.71433 (7)	0.59508 (7)	0.91751 (15)	0.0155 (2)	0.7479 (13)
N3A	0.60604 (7)	0.51973 (7)	0.90775 (16)	0.0166 (3)	0.7479 (13)
H3AB	0.5745	0.5014	0.9620	0.020*	0.7479 (13)
C1A	0.83777 (10)	0.62752 (8)	0.91544 (19)	0.0184 (3)	0.7479 (13)
C2A	0.90681 (10)	0.66651 (10)	0.9555 (2)	0.0250 (4)	0.7479 (13)
H2AA	0.9533	0.6560	0.9185	0.030*	0.7479 (13)
C3A	0.90283 (12)	0.72153 (12)	1.0530 (3)	0.0306 (5)	0.7479 (13)
H3AA	0.9478	0.7478	1.0820	0.037*	0.7479 (13)
C4A	0.83290 (15)	0.73839 (12)	1.1087 (3)	0.0346 (5)	0.7479 (13)
H4AA	0.8324	0.7755	1.1735	0.042*	0.7479 (13)
C5A	0.76429 (11)	0.70022 (11)	1.0683 (3)	0.0296 (4)	0.7479 (13)
H5AA	0.7175	0.7114	1.1036	0.036*	0.7479 (13)
C6A	0.76874 (9)	0.64467 (9)	0.9730 (2)	0.0184 (3)	0.7479 (13)
C7A	0.75068 (11)	0.55190 (12)	0.8291 (2)	0.0153 (3)	0.7479 (13)
C8A	0.70730 (9)	0.49398 (8)	0.75729 (18)	0.0154 (3)	0.7479 (13)
C9A	0.63282 (9)	0.47982 (8)	0.79902 (18)	0.0151 (3)	0.7479 (13)
C10A	0.59061 (10)	0.42188 (10)	0.7374 (2)	0.0208 (3)	0.7479 (13)
H10A	0.5413	0.4117	0.7632	0.025*	0.7479 (13)
C11A	0.62240 (14)	0.37973 (11)	0.6380 (2)	0.0261 (4)	0.7479 (13)
H11A	0.5940	0.3415	0.5976	0.031*	0.7479 (13)
C12A	0.69640 (14)	0.39393 (11)	0.5979 (2)	0.0269 (4)	0.7479 (13)
H12A	0.7173	0.3653	0.5315	0.032*	0.7479 (13)
C13A	0.73847 (10)	0.45117 (10)	0.6579 (2)	0.0212 (3)	0.7479 (13)
H13A	0.7878	0.4610	0.6315	0.025*	0.7479 (13)
C14A	0.63058 (13)	0.59199 (16)	0.9310 (3)	0.0124 (6)	0.7479 (13)
H14A	0.6237	0.6051	1.0308	0.015*	0.7479 (13)
C15A	0.58296 (14)	0.64264 (17)	0.8268 (3)	0.0125 (7)	0.7479 (13)
C16A	0.61480 (14)	0.68116 (17)	0.7214 (3)	0.0201 (6)	0.7479 (13)
H16A	0.6674	0.6753	0.7092	0.024*	0.7479 (13)
C17A	0.5679 (3)	0.7286 (4)	0.6337 (8)	0.0340 (15)	0.7479 (13)
H17A	0.5898	0.7551	0.5650	0.041*	0.7479 (13)
C18A	0.48936 (16)	0.73634 (19)	0.6488 (4)	0.0233 (8)	0.7479 (13)
H18A	0.4583	0.7673	0.5889	0.028*	0.7479 (13)
C19A	0.45662 (18)	0.6981 (2)	0.7532 (5)	0.0178 (9)	0.7479 (13)
H19A	0.4039	0.7041	0.7643	0.021*	0.7479 (13)
C20A	0.50286 (14)	0.65047 (16)	0.8415 (3)	0.0117 (6)	0.7479 (13)
O1B	1.03721 (19)	0.4897 (2)	0.2130 (4)	0.0231 (6)	0.7479 (13)
H1OB	1.0934	0.4797	0.2233	0.028*	0.7479 (13)
N1B	0.67513 (8)	0.41530 (9)	0.15645 (18)	0.0253 (3)	0.7479 (13)
N2B	0.79070 (8)	0.47098 (8)	0.16595 (18)	0.0237 (3)	0.7479 (13)
N3B	0.88892 (9)	0.44281 (9)	0.02448 (18)	0.0261 (3)	0.7479 (13)
H3BB	0.9205	0.4619	−0.0286	0.031*	0.7479 (13)
C1B	0.67384 (10)	0.47560 (10)	0.2429 (2)	0.0243 (3)	0.7479 (13)
C2B	0.61365 (12)	0.50263 (11)	0.3142 (2)	0.0288 (4)	0.7479 (13)
H2BA	0.5655	0.4799	0.3109	0.035*	0.7479 (13)
C3B	0.62839 (19)	0.5640 (2)	0.3893 (5)	0.0256 (7)	0.7479 (13)
H3BA	0.5887	0.5831	0.4362	0.031*	0.7479 (13)
C4B	0.70066 (18)	0.59922 (17)	0.3987 (4)	0.0341 (6)	0.7479 (13)
H4BA	0.7084	0.6404	0.4522	0.041*	0.7479 (13)
C5B	0.76129 (11)	0.57226 (12)	0.3270 (3)	0.0302 (4)	0.7479 (13)
H5BA	0.8096	0.5947	0.3310	0.036*	0.7479 (13)
C6B	0.74580 (12)	0.51085 (10)	0.2504 (2)	0.0233 (3)	0.7479 (13)
C7B	0.74581 (9)	0.41479 (10)	0.1156 (2)	0.0216 (3)	0.7479 (13)
C8B	0.77894 (10)	0.36412 (10)	0.0230 (2)	0.0216 (3)	0.7479 (13)
C9B	0.85229 (10)	0.38073 (10)	−0.0204 (2)	0.0227 (3)	0.7479 (13)
C10B	0.88395 (10)	0.33569 (11)	−0.1166 (2)	0.0266 (4)	0.7479 (13)
H10B	0.9325	0.3457	−0.1461	0.032*	0.7479 (13)
C11B	0.84375 (12)	0.27688 (12)	−0.1674 (3)	0.0265 (4)	0.7479 (13)
H11B	0.8657	0.2480	−0.2321	0.032*	0.7479 (13)
C12B	0.7707 (2)	0.25862 (17)	−0.1257 (4)	0.0244 (6)	0.7479 (13)
H12B	0.7446	0.2182	−0.1606	0.029*	0.7479 (13)
C13B	0.73862 (11)	0.30330 (12)	−0.0298 (3)	0.0262 (4)	0.7479 (13)
H13B	0.6901	0.2927	−0.0005	0.031*	0.7479 (13)
C14B	0.87512 (14)	0.4770 (2)	0.1610 (5)	0.0165 (8)	0.7479 (13)
H14B	0.8893	0.5265	0.1570	0.020*	0.7479 (13)
C15B	0.92214 (18)	0.4436 (2)	0.2945 (4)	0.0144 (10)	0.7479 (13)
C16B	0.88769 (15)	0.40564 (16)	0.3972 (3)	0.0179 (5)	0.7479 (13)
H16B	0.8333	0.4009	0.3868	0.022*	0.7479 (13)
C17B	0.93327 (19)	0.3747 (2)	0.5154 (4)	0.0224 (8)	0.7479 (13)
H17B	0.9094	0.3492	0.5830	0.027*	0.7479 (13)
C18B	1.0145 (2)	0.3818 (2)	0.5324 (5)	0.0211 (7)	0.7479 (13)
H18B	1.0448	0.3617	0.6124	0.025*	0.7479 (13)
C19B	1.0507 (3)	0.4187 (4)	0.4312 (6)	0.0222 (9)	0.7479 (13)
H19B	1.1052	0.4234	0.4422	0.027*	0.7479 (13)
C20B	1.0042 (3)	0.4486 (3)	0.3125 (6)	0.0208 (9)	0.7479 (13)
O1X	0.4731 (8)	0.6132 (8)	0.9421 (16)	0.055 (4)*	0.2521 (13)
H1OX	0.4254	0.6071	0.9301	0.066*	0.2521 (13)
N1X	0.7962 (3)	0.5228 (3)	0.7655 (6)	0.0338 (11)*	0.2521 (13)
N2X	0.6853 (3)	0.5492 (3)	0.8586 (6)	0.0356 (12)*	0.2521 (13)
N3X	0.6784 (3)	0.6299 (3)	1.0464 (7)	0.0429 (14)*	0.2521 (13)
H3XB	0.6602	0.6361	1.1276	0.051*	0.2521 (13)
C1X	0.7358 (3)	0.4754 (3)	0.7172 (7)	0.0264 (11)*	0.2521 (13)
C2X	0.7366 (4)	0.4182 (4)	0.6289 (8)	0.0351 (14)*	0.2521 (13)
H2XA	0.7824	0.4057	0.5914	0.042*	0.2521 (13)
C3X	0.6704 (5)	0.3800 (4)	0.5966 (10)	0.0367 (19)*	0.2521 (13)
H3XA	0.6703	0.3425	0.5327	0.044*	0.2521 (13)
C4X	0.6027 (5)	0.3954 (4)	0.6564 (10)	0.0344 (19)*	0.2521 (13)
H4XA	0.5587	0.3669	0.6360	0.041*	0.2521 (13)
C5X	0.5998 (4)	0.4525 (4)	0.7459 (8)	0.0331 (14)*	0.2521 (13)
H5XA	0.5545	0.4640	0.7856	0.040*	0.2521 (13)
C6X	0.6674 (4)	0.4919 (3)	0.7740 (7)	0.0255 (10)*	0.2521 (13)
C7X	0.7623 (5)	0.5673 (4)	0.8464 (11)	0.034 (3)*	0.2521 (13)
C8X	0.7975 (4)	0.6267 (3)	0.9330 (7)	0.0300 (12)*	0.2521 (13)
C9X	0.7530 (4)	0.6565 (4)	1.0300 (8)	0.0351 (14)*	0.2521 (13)
C10X	0.7850 (5)	0.7112 (4)	1.1162 (10)	0.0420 (19)*	0.2521 (13)
H10C	0.7557	0.7328	1.1810	0.050*	0.2521 (13)
C11X	0.8593 (6)	0.7332 (5)	1.1061 (11)	0.045 (3)*	0.2521 (13)
H11C	0.8804	0.7688	1.1675	0.054*	0.2521 (13)
C12X	0.9049 (5)	0.7050 (5)	1.0090 (9)	0.035 (2)*	0.2521 (13)
H12C	0.9548	0.7223	1.0008	0.042*	0.2521 (13)
C13X	0.8731 (5)	0.6502 (4)	0.9245 (8)	0.0334 (13)*	0.2521 (13)
H13C	0.9029	0.6285	0.8606	0.040*	0.2521 (13)
C14X	0.6312 (8)	0.5922 (9)	0.9299 (16)	0.049 (5)*	0.2521 (13)
H14C	0.5958	0.5608	0.9740	0.058*	0.2521 (13)
C15X	0.5812 (8)	0.6416 (9)	0.8288 (18)	0.044 (5)*	0.2521 (13)
C16X	0.6105 (10)	0.6770 (9)	0.7192 (19)	0.065 (6)*	0.2521 (13)
H16C	0.6634	0.6704	0.7103	0.079*	0.2521 (13)
C17X	0.5687 (11)	0.7214 (11)	0.621 (2)	0.028 (3)*	0.2521 (13)
H17C	0.5901	0.7410	0.5436	0.033*	0.2521 (13)
C18X	0.4932 (10)	0.7351 (11)	0.647 (2)	0.063 (6)*	0.2521 (13)
H18C	0.4646	0.7695	0.5919	0.076*	0.2521 (13)
C19X	0.4586 (10)	0.6994 (11)	0.750 (2)	0.046 (6)*	0.2521 (13)
H19C	0.4061	0.7072	0.7602	0.055*	0.2521 (13)
C20X	0.5018 (8)	0.6524 (10)	0.838 (2)	0.048 (5)*	0.2521 (13)
O1Y	1.0330 (7)	0.4857 (7)	0.1895 (12)	0.024 (2)*	0.2521 (13)
H1OY	1.0840	0.4785	0.2002	0.028*	0.2521 (13)
N1Y	0.6910 (2)	0.38802 (19)	0.0948 (4)	0.0128 (7)*	0.2521 (13)
N2Y	0.8087 (2)	0.43856 (19)	0.1044 (4)	0.0132 (7)*	0.2521 (13)
N3Y	0.8417 (2)	0.5488 (2)	0.2063 (4)	0.0174 (7)*	0.2521 (13)
H3YB	0.8655	0.5872	0.1907	0.021*	0.2521 (13)
C1Y	0.7373 (2)	0.3489 (2)	0.0136 (5)	0.0116 (7)*	0.2521 (13)
C2Y	0.7211 (4)	0.2875 (3)	−0.0681 (7)	0.0241 (14)*	0.2521 (13)
H2YA	0.6729	0.2645	−0.0750	0.029*	0.2521 (13)
C3Y	0.7835 (6)	0.2639 (6)	−0.1384 (14)	0.025 (3)*	0.2521 (13)
H3YA	0.7748	0.2245	−0.1974	0.030*	0.2521 (13)
C4Y	0.8562 (4)	0.2940 (3)	−0.1270 (7)	0.0218 (13)*	0.2521 (13)
H4YA	0.8954	0.2732	−0.1727	0.026*	0.2521 (13)
C5Y	0.8719 (3)	0.3535 (3)	−0.0503 (6)	0.0152 (8)*	0.2521 (13)
H5YA	0.9206	0.3754	−0.0448	0.018*	0.2521 (13)
C6Y	0.8125 (3)	0.3803 (2)	0.0192 (5)	0.0105 (7)*	0.2521 (13)
C7Y	0.7352 (3)	0.4417 (2)	0.1501 (5)	0.0138 (8)*	0.2521 (13)
C8Y	0.7164 (3)	0.4979 (2)	0.2431 (5)	0.0131 (9)*	0.2521 (13)
C9Y	0.7740 (3)	0.5507 (3)	0.2677 (6)	0.0195 (9)*	0.2521 (13)
C10Y	0.7561 (3)	0.6108 (3)	0.3521 (6)	0.0258 (10)*	0.2521 (13)
H10D	0.7921	0.6472	0.3698	0.031*	0.2521 (13)
C11Y	0.6862 (6)	0.6133 (5)	0.4052 (14)	0.036 (3)*	0.2521 (13)
H11D	0.6745	0.6529	0.4565	0.043*	0.2521 (13)
C12Y	0.6304 (11)	0.5587 (9)	0.386 (3)	0.056 (4)*	0.2521 (13)
H12D	0.5845	0.5603	0.4297	0.067*	0.2521 (13)
C13Y	0.6468 (3)	0.5023 (3)	0.3001 (6)	0.0192 (9)*	0.2521 (13)
H13D	0.6097	0.4668	0.2808	0.023*	0.2521 (13)
C14Y	0.8749 (8)	0.4810 (7)	0.166 (2)	0.041 (5)*	0.2521 (13)
H14D	0.9078	0.4900	0.0892	0.050*	0.2521 (13)
C15Y	0.9251 (8)	0.4442 (10)	0.2900 (19)	0.036 (6)*	0.2521 (13)
C16Y	0.8933 (9)	0.4054 (9)	0.3928 (19)	0.056 (4)*	0.2521 (13)
H16D	0.8392	0.3985	0.3813	0.067*	0.2521 (13)
C17Y	0.9389 (10)	0.3760 (11)	0.514 (2)	0.057 (6)*	0.2521 (13)
H17D	0.9171	0.3522	0.5862	0.069*	0.2521 (13)
C18Y	1.0187 (10)	0.3851 (11)	0.517 (2)	0.046 (5)*	0.2521 (13)
H18D	1.0524	0.3627	0.5895	0.055*	0.2521 (13)
C19Y	1.0504 (10)	0.4253 (12)	0.419 (2)	0.026 (4)*	0.2521 (13)
H19D	1.1047	0.4316	0.4304	0.031*	0.2521 (13)
C20Y	1.0060 (7)	0.4567 (8)	0.3046 (14)	0.0095 (19)*	0.2521 (13)

### Atomic displacement parameters (Å^2^)

**Table:**

	*U*^11^	*U*^22^	*U*^33^	*U*^12^	*U*^13^	*U*^23^
O1A	0.0074 (5)	0.0250 (8)	0.0163 (7)	0.0011 (4)	0.0035 (3)	0.0049 (5)
N1A	0.0115 (5)	0.0228 (6)	0.0231 (7)	0.0008 (4)	0.0033 (4)	0.0029 (6)
N2A	0.0098 (5)	0.0170 (5)	0.0197 (6)	0.0012 (4)	0.0019 (4)	−0.0012 (5)
N3A	0.0134 (5)	0.0164 (5)	0.0212 (7)	−0.0001 (4)	0.0070 (4)	0.0019 (5)
C1A	0.0105 (6)	0.0198 (7)	0.0243 (8)	−0.0009 (5)	0.0008 (5)	0.0052 (6)
C2A	0.0134 (7)	0.0249 (8)	0.0350 (10)	−0.0046 (6)	−0.0030 (6)	0.0057 (8)
C3A	0.0214 (9)	0.0263 (10)	0.0410 (13)	−0.0029 (7)	−0.0069 (8)	−0.0022 (10)
C4A	0.0244 (10)	0.0264 (10)	0.0504 (15)	0.0004 (8)	−0.0053 (9)	−0.0125 (9)
C5A	0.0179 (7)	0.0259 (9)	0.0428 (12)	0.0018 (6)	−0.0037 (8)	−0.0117 (9)
C6A	0.0130 (6)	0.0198 (7)	0.0220 (8)	0.0015 (5)	0.0005 (6)	−0.0020 (6)
C7A	0.0113 (6)	0.0175 (8)	0.0176 (8)	0.0030 (7)	0.0040 (6)	0.0033 (7)
C8A	0.0118 (6)	0.0172 (6)	0.0179 (7)	0.0027 (5)	0.0047 (5)	0.0005 (6)
C9A	0.0128 (6)	0.0154 (6)	0.0175 (7)	0.0001 (5)	0.0032 (5)	0.0032 (6)
C10A	0.0199 (7)	0.0184 (8)	0.0239 (9)	−0.0046 (6)	0.0021 (6)	0.0003 (7)
C11A	0.0342 (10)	0.0186 (8)	0.0261 (10)	−0.0038 (8)	0.0063 (8)	−0.0036 (7)
C12A	0.0318 (10)	0.0218 (8)	0.0287 (10)	0.0001 (8)	0.0100 (8)	−0.0042 (7)
C13A	0.0229 (7)	0.0196 (8)	0.0223 (8)	0.0020 (6)	0.0075 (6)	−0.0037 (7)
C14A	0.0073 (7)	0.0168 (9)	0.0134 (9)	0.0026 (4)	0.0027 (4)	0.0008 (5)
C15A	0.0076 (7)	0.0157 (9)	0.0141 (10)	0.0018 (4)	0.0012 (4)	0.0005 (5)
C16A	0.0084 (6)	0.0285 (10)	0.0235 (10)	0.0003 (5)	0.0031 (5)	0.0113 (7)
C17A	0.0188 (11)	0.040 (3)	0.043 (3)	−0.0027 (11)	0.0015 (10)	0.024 (2)
C18A	0.0134 (7)	0.0226 (10)	0.0319 (13)	0.0004 (5)	−0.0042 (6)	0.0144 (8)
C19A	0.0090 (8)	0.0189 (11)	0.0244 (13)	0.0020 (5)	−0.0017 (5)	0.0021 (6)
C20A	0.0090 (7)	0.0133 (8)	0.0127 (9)	0.0005 (4)	0.0006 (4)	−0.0006 (5)
O1B	0.0110 (7)	0.0381 (13)	0.0213 (12)	0.0031 (6)	0.0060 (8)	0.0082 (11)
N1B	0.0152 (6)	0.0329 (8)	0.0274 (8)	−0.0009 (5)	0.0016 (5)	0.0018 (7)
N2B	0.0157 (6)	0.0257 (7)	0.0298 (8)	0.0000 (5)	0.0032 (5)	0.0058 (6)
N3B	0.0201 (6)	0.0335 (8)	0.0257 (8)	−0.0034 (5)	0.0070 (5)	0.0063 (7)
C1B	0.0165 (7)	0.0318 (9)	0.0246 (9)	0.0031 (6)	0.0022 (6)	0.0032 (7)
C2B	0.0180 (8)	0.0399 (11)	0.0288 (10)	0.0018 (7)	0.0036 (7)	−0.0006 (8)
C3B	0.0170 (8)	0.0360 (12)	0.0236 (10)	0.0059 (7)	0.0023 (6)	−0.0044 (8)
C4B	0.0307 (12)	0.0370 (14)	0.0336 (13)	−0.0007 (11)	0.0004 (10)	−0.0056 (11)
C5B	0.0219 (8)	0.0321 (10)	0.0360 (11)	−0.0007 (7)	0.0013 (7)	0.0034 (9)
C6B	0.0176 (8)	0.0260 (8)	0.0261 (9)	0.0010 (7)	0.0025 (6)	0.0043 (7)
C7B	0.0149 (6)	0.0263 (8)	0.0230 (8)	−0.0002 (6)	0.0002 (6)	0.0066 (7)
C8B	0.0169 (8)	0.0257 (8)	0.0224 (8)	0.0021 (6)	0.0027 (6)	0.0057 (7)
C9B	0.0175 (7)	0.0288 (9)	0.0216 (8)	0.0021 (6)	0.0024 (6)	0.0056 (7)
C10B	0.0192 (7)	0.0368 (10)	0.0238 (9)	0.0016 (7)	0.0025 (6)	0.0036 (8)
C11B	0.0220 (8)	0.0311 (10)	0.0263 (10)	0.0047 (7)	0.0029 (7)	0.0019 (9)
C12B	0.0187 (11)	0.0263 (11)	0.0281 (13)	−0.0032 (9)	0.0028 (10)	0.0016 (9)
C13B	0.0187 (8)	0.0318 (10)	0.0284 (10)	0.0026 (7)	0.0047 (7)	0.0048 (9)
C14B	0.0076 (8)	0.0203 (10)	0.0216 (12)	−0.0010 (5)	0.0027 (5)	0.0065 (7)
C15B	0.0099 (9)	0.0158 (12)	0.0177 (13)	−0.0001 (5)	0.0023 (5)	0.0022 (6)
C16B	0.0110 (7)	0.0216 (9)	0.0212 (9)	−0.0043 (5)	0.0019 (5)	0.0069 (6)
C17B	0.0165 (8)	0.0280 (12)	0.0227 (12)	−0.0035 (6)	0.0017 (6)	0.0109 (7)
C18B	0.0177 (10)	0.0263 (13)	0.0188 (11)	0.0033 (6)	0.0007 (7)	0.0082 (9)
C19B	0.0140 (10)	0.0296 (19)	0.0228 (16)	0.0054 (8)	0.0019 (7)	0.0060 (11)
C20B	0.0186 (11)	0.0198 (18)	0.0254 (14)	0.0007 (9)	0.0084 (7)	0.0045 (10)

### Geometric parameters (Å, °)

**Table:**

O1A—C20A	1.361 (3)	O1X—C20X	1.362 (13)
O1A—H1OA	0.9812	O1X—H1OX	0.8201
N1A—C7A	1.325 (2)	N1X—C7X	1.318 (9)
N1A—C1A	1.387 (2)	N1X—C1X	1.405 (7)
N2A—C7A	1.370 (2)	N2X—C6X	1.358 (7)
N2A—C6A	1.381 (2)	N2X—C7X	1.385 (9)
N2A—C14A	1.460 (3)	N2X—C14X	1.461 (12)
N3A—C9A	1.389 (2)	N3X—C9X	1.404 (8)
N3A—C14A	1.452 (3)	N3X—C14X	1.453 (15)
N3A—H3AB	0.8600	N3X—H3XB	0.8600
C1A—C6A	1.400 (2)	C1X—C2X	1.367 (9)
C1A—C2A	1.407 (2)	C1X—C6X	1.383 (9)
C2A—C3A	1.394 (3)	C2X—C3X	1.350 (9)
C2A—H2AA	0.9300	C2X—H2XA	0.9300
C3A—C4A	1.404 (4)	C3X—C4X	1.383 (10)
C3A—H3AA	0.9300	C3X—H3XA	0.9300
C4A—C5A	1.393 (3)	C4X—C5X	1.375 (10)
C4A—H4AA	0.9300	C4X—H4XA	0.9300
C5A—C6A	1.390 (3)	C5X—C6X	1.378 (9)
C5A—H5AA	0.9300	C5X—H5XA	0.9300
C7A—C8A	1.446 (3)	C7X—C8X	1.473 (9)
C8A—C13A	1.390 (2)	C8X—C9X	1.376 (9)
C8A—C9A	1.409 (2)	C8X—C13X	1.384 (9)
C9A—C10A	1.403 (2)	C9X—C10X	1.385 (10)
C10A—C11A	1.388 (3)	C10X—C11X	1.356 (11)
C10A—H10A	0.9300	C10X—H10C	0.9300
C11A—C12A	1.395 (3)	C11X—C12X	1.377 (11)
C11A—H11A	0.9300	C11X—H11C	0.9300
C12A—C13A	1.386 (3)	C12X—C13X	1.378 (10)
C12A—H12A	0.9300	C12X—H12C	0.9300
C13A—H13A	0.9300	C13X—H13C	0.9300
C14A—C15A	1.526 (3)	C14X—C15X	1.514 (14)
C14A—H14A	0.9800	C14X—H14C	0.9800
C15A—C16A	1.391 (3)	C15X—C16X	1.370 (14)
C15A—C20A	1.406 (3)	C15X—C20X	1.392 (13)
C16A—C17A	1.399 (5)	C16X—C17X	1.372 (14)
C16A—H16A	0.9300	C16X—H16C	0.9300
C17A—C18A	1.381 (5)	C17X—C18X	1.371 (15)
C17A—H17A	0.9300	C17X—H17C	0.9300
C18A—C19A	1.389 (3)	C18X—C19X	1.375 (14)
C18A—H18A	0.9300	C18X—H18C	0.9300
C19A—C20A	1.398 (3)	C19X—C20X	1.364 (14)
C19A—H19A	0.9300	C19X—H19C	0.9300
O1B—C20B	1.387 (4)	O1Y—C20Y	1.337 (12)
O1B—H1OB	0.9754	O1Y—H1OY	0.8786
N1B—C7B	1.317 (2)	N1Y—C7Y	1.337 (6)
N1B—C1B	1.405 (3)	N1Y—C1Y	1.382 (5)
N2B—C7B	1.367 (2)	N2Y—C6Y	1.371 (5)
N2B—C6B	1.395 (2)	N2Y—C7Y	1.384 (6)
N2B—C14B	1.459 (3)	N2Y—C14Y	1.451 (12)
N3B—C9B	1.381 (2)	N3Y—C9Y	1.359 (6)
N3B—C14B	1.470 (5)	N3Y—C14Y	1.483 (16)
N3B—H3BB	0.8600	N3Y—H3YB	0.8600
C1B—C2B	1.396 (3)	C1Y—C2Y	1.404 (7)
C1B—C6B	1.400 (3)	C1Y—C6Y	1.417 (7)
C2B—C3B	1.369 (4)	C2Y—C3Y	1.399 (11)
C2B—H2BA	0.9300	C2Y—H2YA	0.9300
C3B—C4B	1.403 (4)	C3Y—C4Y	1.364 (10)
C3B—H3BA	0.9300	C3Y—H3YA	0.9300
C4B—C5B	1.404 (4)	C4Y—C5Y	1.350 (8)
C4B—H4BA	0.9300	C4Y—H4YA	0.9300
C5B—C6B	1.379 (3)	C5Y—C6Y	1.374 (7)
C5B—H5BA	0.9300	C5Y—H5YA	0.9300
C7B—C8B	1.458 (3)	C7Y—C8Y	1.439 (6)
C8B—C9B	1.407 (2)	C8Y—C13Y	1.371 (7)
C8B—C13B	1.407 (3)	C8Y—C9Y	1.409 (7)
C9B—C10B	1.399 (3)	C9Y—C10Y	1.445 (8)
C10B—C11B	1.370 (3)	C10Y—C11Y	1.356 (10)
C10B—H10B	0.9300	C10Y—H10D	0.9300
C11B—C12B	1.403 (4)	C11Y—C12Y	1.412 (16)
C11B—H11B	0.9300	C11Y—H11D	0.9300
C12B—C13B	1.397 (4)	C12Y—C13Y	1.393 (15)
C12B—H12B	0.9300	C12Y—H12D	0.9300
C13B—H13B	0.9300	C13Y—H13D	0.9300
C14B—C15B	1.524 (3)	C14Y—C15Y	1.515 (14)
C14B—H14B	0.9800	C14Y—H14D	0.9800
C15B—C16B	1.389 (3)	C15Y—C16Y	1.376 (14)
C15B—C20B	1.398 (4)	C15Y—C20Y	1.396 (13)
C16B—C17B	1.390 (3)	C16Y—C17Y	1.397 (14)
C16B—H16B	0.9300	C16Y—H16D	0.9300
C17B—C18B	1.389 (4)	C17Y—C18Y	1.376 (14)
C17B—H17B	0.9300	C17Y—H17D	0.9300
C18B—C19B	1.384 (5)	C18Y—C19Y	1.356 (14)
C18B—H18B	0.9300	C18Y—H18D	0.9300
C19B—C20B	1.394 (5)	C19Y—C20Y	1.360 (13)
C19B—H19B	0.9300	C19Y—H19D	0.9300
			
C20A—O1A—H1OA	113.6	C20X—O1X—H1OA	114.8
C20A—O1A—H1OX	113.8	C20X—O1X—H1OX	115.4
C7A—N1A—C1A	105.22 (16)	C7X—N1X—C1X	103.6 (6)
C7A—N2A—C6A	107.32 (15)	C6X—N2X—C7X	107.4 (6)
C7A—N2A—C14A	123.90 (18)	C6X—N2X—C14X	127.3 (8)
C6A—N2A—C14A	128.34 (16)	C7X—N2X—C14X	124.5 (8)
C9A—N3A—C14A	120.86 (15)	C9X—N3X—C14X	121.7 (8)
C9A—N3A—H3AB	119.6	C9X—N3X—H3XB	119.1
C14A—N3A—H3AB	119.6	C14X—N3X—H3XB	119.1
N1A—C1A—C6A	110.06 (15)	C2X—C1X—C6X	119.1 (6)
N1A—C1A—C2A	129.92 (16)	C2X—C1X—N1X	129.9 (6)
C6A—C1A—C2A	120.00 (16)	C6X—C1X—N1X	111.0 (6)
C3A—C2A—C1A	117.34 (17)	C3X—C2X—C1X	119.3 (7)
C3A—C2A—H2AA	121.3	C3X—C2X—H2XA	120.3
C1A—C2A—H2AA	121.3	C1X—C2X—H2XA	120.3
C2A—C3A—C4A	121.92 (18)	C2X—C3X—C4X	121.5 (8)
C2A—C3A—H3AA	119.0	C2X—C3X—H3XA	119.2
C4A—C3A—H3AA	119.0	C4X—C3X—H3XA	119.2
C5A—C4A—C3A	120.9 (2)	C5X—C4X—C3X	120.6 (7)
C5A—C4A—H4AA	119.6	C5X—C4X—H4XA	119.7
C3A—C4A—H4AA	119.6	C3X—C4X—H4XA	119.7
C6A—C5A—C4A	117.10 (19)	C4X—C5X—C6X	116.8 (6)
C6A—C5A—H5AA	121.5	C4X—C5X—H5XA	121.6
C4A—C5A—H5AA	121.5	C6X—C5X—H5XA	121.6
N2A—C6A—C5A	132.06 (17)	N2X—C6X—C5X	132.0 (7)
N2A—C6A—C1A	105.16 (15)	N2X—C6X—C1X	105.4 (6)
C5A—C6A—C1A	122.76 (16)	C5X—C6X—C1X	122.5 (6)
N1A—C7A—N2A	112.2 (2)	N1X—C7X—N2X	112.5 (6)
N1A—C7A—C8A	128.45 (17)	N1X—C7X—C8X	128.8 (7)
N2A—C7A—C8A	119.30 (16)	N2X—C7X—C8X	118.3 (7)
C13A—C8A—C9A	120.70 (15)	C9X—C8X—C13X	120.6 (6)
C13A—C8A—C7A	122.39 (15)	C9X—C8X—C7X	116.8 (7)
C9A—C8A—C7A	116.81 (15)	C13X—C8X—C7X	122.5 (7)
N3A—C9A—C10A	121.76 (15)	C8X—C9X—C10X	118.5 (6)
N3A—C9A—C8A	119.48 (14)	C8X—C9X—N3X	120.9 (7)
C10A—C9A—C8A	118.55 (15)	C10X—C9X—N3X	120.6 (7)
C11A—C10A—C9A	120.09 (16)	C11X—C10X—C9X	119.9 (8)
C11A—C10A—H10A	120.0	C11X—C10X—H10C	120.0
C9A—C10A—H10A	120.0	C9X—C10X—H10C	120.0
C10A—C11A—C12A	120.97 (18)	C10X—C11X—C12X	122.9 (9)
C10A—C11A—H11A	119.5	C10X—C11X—H11C	118.5
C12A—C11A—H11A	119.5	C12X—C11X—H11C	118.5
C13A—C12A—C11A	119.37 (18)	C11X—C12X—C13X	116.9 (8)
C13A—C12A—H12A	120.3	C11X—C12X—H12C	121.5
C11A—C12A—H12A	120.3	C13X—C12X—H12C	121.5
C12A—C13A—C8A	120.32 (16)	C12X—C13X—C8X	121.1 (7)
C12A—C13A—H13A	119.8	C12X—C13X—H13C	119.4
C8A—C13A—H13A	119.8	C8X—C13X—H13C	119.4
N3A—C14A—N2A	107.16 (16)	N3X—C14X—N2X	106.8 (9)
N3A—C14A—C15A	112.9 (2)	N3X—C14X—C15X	111.3 (13)
N2A—C14A—C15A	111.8 (2)	N2X—C14X—C15X	114.4 (11)
N3A—C14A—H14A	108.3	N3X—C14X—H14C	108.0
N2A—C14A—H14A	108.3	N2X—C14X—H14C	108.0
C15A—C14A—H14A	108.3	C15X—C14X—H14C	108.0
C16A—C15A—C20A	119.4 (2)	C16X—C15X—C20X	115.6 (12)
C16A—C15A—C14A	123.6 (2)	C16X—C15X—C14X	122.2 (13)
C20A—C15A—C14A	117.0 (2)	C20X—C15X—C14X	122.2 (12)
C15A—C16A—C17A	120.1 (3)	C15X—C16X—C17X	125.7 (15)
C15A—C16A—H16A	119.9	C15X—C16X—H16C	117.2
C17A—C16A—H16A	119.9	C17X—C16X—H16C	117.2
C18A—C17A—C16A	120.3 (4)	C18X—C17X—C16X	115.4 (15)
C18A—C17A—H17A	119.9	C18X—C17X—H17C	122.3
C16A—C17A—H17A	119.9	C16X—C17X—H17C	122.3
C17A—C18A—C19A	120.3 (3)	C17X—C18X—C19X	122.0 (15)
C17A—C18A—H18A	119.9	C17X—C18X—H18C	119.0
C19A—C18A—H18A	119.9	C19X—C18X—H18C	119.0
C18A—C19A—C20A	120.0 (3)	C20X—C19X—C18X	119.6 (15)
C18A—C19A—H19A	120.0	C20X—C19X—H19C	120.2
C20A—C19A—H19A	120.0	C18X—C19X—H19C	120.2
O1A—C20A—C19A	122.0 (2)	O1X—C20X—C19X	124.6 (14)
O1A—C20A—C15A	118.1 (2)	O1X—C20X—C15X	114.2 (13)
C19A—C20A—C15A	119.9 (2)	C19X—C20X—C15X	121.2 (13)
C20B—O1B—H1OB	108.0	C20Y—O1Y—H1OB	98.5
C20B—O1B—H1OY	115.2	C20Y—O1Y—H1OY	106.8
C7B—N1B—C1B	104.73 (15)	C7Y—N1Y—C1Y	106.7 (4)
C7B—N2B—C6B	107.03 (15)	C6Y—N2Y—C7Y	109.0 (4)
C7B—N2B—C14B	124.6 (2)	C6Y—N2Y—C14Y	125.9 (8)
C6B—N2B—C14B	126.8 (2)	C7Y—N2Y—C14Y	123.6 (8)
C9B—N3B—C14B	121.47 (17)	C9Y—N3Y—C14Y	120.4 (7)
C9B—N3B—H3BB	119.3	C9Y—N3Y—H3YB	119.8
C14B—N3B—H3BB	119.3	C14Y—N3Y—H3YB	119.8
C2B—C1B—C6B	120.02 (18)	N1Y—C1Y—C2Y	131.2 (4)
C2B—C1B—N1B	129.91 (17)	N1Y—C1Y—C6Y	109.6 (4)
C6B—C1B—N1B	110.07 (15)	C2Y—C1Y—C6Y	119.2 (4)
C3B—C2B—C1B	117.3 (2)	C3Y—C2Y—C1Y	114.3 (6)
C3B—C2B—H2BA	121.3	C3Y—C2Y—H2YA	122.8
C1B—C2B—H2BA	121.3	C1Y—C2Y—H2YA	122.8
C2B—C3B—C4B	123.0 (3)	C4Y—C3Y—C2Y	125.1 (9)
C2B—C3B—H3BA	118.5	C4Y—C3Y—H3YA	117.4
C4B—C3B—H3BA	118.5	C2Y—C3Y—H3YA	117.4
C3B—C4B—C5B	119.9 (3)	C5Y—C4Y—C3Y	120.9 (7)
C3B—C4B—H4BA	120.1	C5Y—C4Y—H4YA	119.6
C5B—C4B—H4BA	120.1	C3Y—C4Y—H4YA	119.6
C6B—C5B—C4B	116.8 (2)	C4Y—C5Y—C6Y	116.9 (5)
C6B—C5B—H5BA	121.6	C4Y—C5Y—H5YA	121.6
C4B—C5B—H5BA	121.6	C6Y—C5Y—H5YA	121.6
C5B—C6B—N2B	132.11 (19)	N2Y—C6Y—C5Y	131.8 (4)
C5B—C6B—C1B	122.96 (18)	N2Y—C6Y—C1Y	104.7 (4)
N2B—C6B—C1B	104.92 (17)	C5Y—C6Y—C1Y	123.5 (4)
N1B—C7B—N2B	113.23 (18)	N1Y—C7Y—N2Y	110.0 (4)
N1B—C7B—C8B	128.25 (17)	N1Y—C7Y—C8Y	130.0 (4)
N2B—C7B—C8B	118.49 (15)	N2Y—C7Y—C8Y	120.0 (4)
C9B—C8B—C13B	120.29 (17)	C13Y—C8Y—C9Y	121.5 (4)
C9B—C8B—C7B	116.80 (17)	C13Y—C8Y—C7Y	123.4 (5)
C13B—C8B—C7B	122.79 (16)	C9Y—C8Y—C7Y	115.1 (5)
N3B—C9B—C10B	121.40 (16)	N3Y—C9Y—C8Y	122.1 (5)
N3B—C9B—C8B	119.77 (18)	N3Y—C9Y—C10Y	120.2 (4)
C10B—C9B—C8B	118.70 (17)	C8Y—C9Y—C10Y	117.5 (5)
C11B—C10B—C9B	120.27 (17)	C11Y—C10Y—C9Y	119.1 (6)
C11B—C10B—H10B	119.9	C11Y—C10Y—H10D	120.4
C9B—C10B—H10B	119.9	C9Y—C10Y—H10D	120.4
C10B—C11B—C12B	122.4 (2)	C10Y—C11Y—C12Y	123.1 (10)
C10B—C11B—H11B	118.8	C10Y—C11Y—H11D	118.4
C12B—C11B—H11B	118.8	C12Y—C11Y—H11D	118.4
C13B—C12B—C11B	117.7 (3)	C13Y—C12Y—C11Y	117.3 (13)
C13B—C12B—H12B	121.2	C13Y—C12Y—H12D	121.4
C11B—C12B—H12B	121.2	C11Y—C12Y—H12D	121.4
C12B—C13B—C8B	120.6 (2)	C8Y—C13Y—C12Y	121.3 (8)
C12B—C13B—H13B	119.7	C8Y—C13Y—H13D	119.3
C8B—C13B—H13B	119.7	C12Y—C13Y—H13D	119.3
N2B—C14B—N3B	105.4 (2)	N2Y—C14Y—N3Y	106.3 (9)
N2B—C14B—C15B	111.3 (3)	N2Y—C14Y—C15Y	112.1 (11)
N3B—C14B—C15B	112.6 (3)	N3Y—C14Y—C15Y	114.5 (15)
N2B—C14B—H14B	109.2	N2Y—C14Y—H14D	107.9
N3B—C14B—H14B	109.2	N3Y—C14Y—H14D	107.9
C15B—C14B—H14B	109.2	C15Y—C14Y—H14D	107.9
C16B—C15B—C20B	117.8 (3)	C16Y—C15Y—C20Y	120.4 (12)
C16B—C15B—C14B	123.2 (3)	C16Y—C15Y—C14Y	122.5 (13)
C20B—C15B—C14B	119.0 (3)	C20Y—C15Y—C14Y	116.9 (11)
C15B—C16B—C17B	121.1 (2)	C15Y—C16Y—C17Y	122.7 (14)
C15B—C16B—H16B	119.5	C15Y—C16Y—H16D	118.7
C17B—C16B—H16B	119.5	C17Y—C16Y—H16D	118.7
C18B—C17B—C16B	119.9 (3)	C18Y—C17Y—C16Y	114.7 (14)
C18B—C17B—H17B	120.0	C18Y—C17Y—H17D	122.7
C16B—C17B—H17B	120.0	C16Y—C17Y—H17D	122.7
C19B—C18B—C17B	120.4 (3)	C19Y—C18Y—C17Y	122.8 (15)
C19B—C18B—H18B	119.8	C19Y—C18Y—H18D	118.6
C17B—C18B—H18B	119.8	C17Y—C18Y—H18D	118.6
C18B—C19B—C20B	118.8 (4)	C18Y—C19Y—C20Y	122.6 (15)
C18B—C19B—H19B	120.6	C18Y—C19Y—H19D	118.7
C20B—C19B—H19B	120.6	C20Y—C19Y—H19D	118.7
O1B—C20B—C19B	121.0 (4)	O1Y—C20Y—C19Y	126.0 (12)
O1B—C20B—C15B	116.9 (4)	O1Y—C20Y—C15Y	115.9 (11)
C19B—C20B—C15B	121.9 (4)	C19Y—C20Y—C15Y	116.5 (12)
			
C7A—N1A—C1A—C6A	−0.70 (19)	C7X—N1X—C1X—C2X	179.6 (8)
C7A—N1A—C1A—C2A	177.60 (18)	C7X—N1X—C1X—C6X	−1.6 (8)
N1A—C1A—C2A—C3A	−178.11 (18)	C6X—C1X—C2X—C3X	1.4 (10)
C6A—C1A—C2A—C3A	0.0 (3)	N1X—C1X—C2X—C3X	−179.8 (7)
C1A—C2A—C3A—C4A	−0.6 (3)	C1X—C2X—C3X—C4X	−3.1 (12)
C2A—C3A—C4A—C5A	0.1 (4)	C2X—C3X—C4X—C5X	3.0 (13)
C3A—C4A—C5A—C6A	0.9 (4)	C3X—C4X—C5X—C6X	−1.1 (12)
C7A—N2A—C6A—C5A	−179.3 (2)	C7X—N2X—C6X—C5X	−180.0 (8)
C14A—N2A—C6A—C5A	8.2 (3)	C14X—N2X—C6X—C5X	−9.8 (13)
C7A—N2A—C6A—C1A	−1.11 (18)	C7X—N2X—C6X—C1X	2.2 (8)
C14A—N2A—C6A—C1A	−173.61 (19)	C14X—N2X—C6X—C1X	172.4 (9)
C4A—C5A—C6A—N2A	176.4 (2)	C4X—C5X—C6X—N2X	−178.1 (7)
C4A—C5A—C6A—C1A	−1.5 (3)	C4X—C5X—C6X—C1X	−0.6 (10)
N1A—C1A—C6A—N2A	1.13 (18)	C2X—C1X—C6X—N2X	178.5 (6)
C2A—C1A—C6A—N2A	−177.36 (15)	N1X—C1X—C6X—N2X	−0.5 (7)
N1A—C1A—C6A—C5A	179.53 (17)	C2X—C1X—C6X—C5X	0.5 (10)
C2A—C1A—C6A—C5A	1.0 (3)	N1X—C1X—C6X—C5X	−178.5 (6)
C1A—N1A—C7A—N2A	0.0 (2)	C1X—N1X—C7X—N2X	3.0 (9)
C1A—N1A—C7A—C8A	−178.26 (18)	C1X—N1X—C7X—C8X	176.3 (9)
C6A—N2A—C7A—N1A	0.7 (2)	C6X—N2X—C7X—N1X	−3.5 (10)
C14A—N2A—C7A—N1A	173.66 (18)	C14X—N2X—C7X—N1X	−174.0 (9)
C6A—N2A—C7A—C8A	179.15 (15)	C6X—N2X—C7X—C8X	−177.6 (7)
C14A—N2A—C7A—C8A	−7.9 (3)	C14X—N2X—C7X—C8X	11.9 (14)
N1A—C7A—C8A—C13A	−6.7 (3)	N1X—C7X—C8X—C9X	−167.6 (9)
N2A—C7A—C8A—C13A	175.20 (16)	N2X—C7X—C8X—C9X	5.4 (12)
N1A—C7A—C8A—C9A	169.64 (17)	N1X—C7X—C8X—C13X	9.2 (14)
N2A—C7A—C8A—C9A	−8.5 (2)	N2X—C7X—C8X—C13X	−177.8 (7)
C14A—N3A—C9A—C10A	−155.79 (17)	C13X—C8X—C9X—C10X	1.4 (11)
C14A—N3A—C9A—C8A	29.6 (2)	C7X—C8X—C9X—C10X	178.2 (8)
C13A—C8A—C9A—N3A	174.43 (15)	C13X—C8X—C9X—N3X	−175.6 (6)
C7A—C8A—C9A—N3A	−2.0 (2)	C7X—C8X—C9X—N3X	1.2 (10)
C13A—C8A—C9A—C10A	−0.4 (2)	C14X—N3X—C9X—C8X	−25.8 (12)
C7A—C8A—C9A—C10A	−176.78 (15)	C14X—N3X—C9X—C10X	157.3 (9)
N3A—C9A—C10A—C11A	−174.50 (17)	C8X—C9X—C10X—C11X	−1.5 (12)
C8A—C9A—C10A—C11A	0.2 (3)	N3X—C9X—C10X—C11X	175.5 (8)
C9A—C10A—C11A—C12A	0.1 (3)	C9X—C10X—C11X—C12X	2.3 (15)
C10A—C11A—C12A—C13A	−0.3 (3)	C10X—C11X—C12X—C13X	−2.9 (14)
C11A—C12A—C13A—C8A	0.1 (3)	C11X—C12X—C13X—C8X	2.7 (12)
C9A—C8A—C13A—C12A	0.2 (3)	C9X—C8X—C13X—C12X	−2.1 (11)
C7A—C8A—C13A—C12A	176.42 (18)	C7X—C8X—C13X—C12X	−178.8 (8)
C9A—N3A—C14A—N2A	−41.4 (2)	C9X—N3X—C14X—N2X	38.1 (14)
C9A—N3A—C14A—C15A	82.0 (2)	C9X—N3X—C14X—C15X	−87.5 (11)
C7A—N2A—C14A—N3A	30.8 (3)	C6X—N2X—C14X—N3X	159.8 (8)
C6A—N2A—C14A—N3A	−157.78 (16)	C7X—N2X—C14X—N3X	−31.6 (15)
C7A—N2A—C14A—C15A	−93.4 (3)	C6X—N2X—C14X—C15X	−76.5 (16)
C6A—N2A—C14A—C15A	78.0 (3)	C7X—N2X—C14X—C15X	92.1 (14)
N3A—C14A—C15A—C16A	−114.4 (3)	N3X—C14X—C15X—C16X	81.8 (17)
N2A—C14A—C15A—C16A	6.5 (4)	N2X—C14X—C15X—C16X	−39 (2)
N3A—C14A—C15A—C20A	66.4 (3)	N3X—C14X—C15X—C20X	−100.2 (19)
N2A—C14A—C15A—C20A	−172.7 (3)	N2X—C14X—C15X—C20X	138.5 (17)
C20A—C15A—C16A—C17A	1.8 (5)	C20X—C15X—C16X—C17X	0.4 (18)
C14A—C15A—C16A—C17A	−177.5 (5)	C14X—C15X—C16X—C17X	178.5 (19)
C15A—C16A—C17A—C18A	−1.6 (8)	C15X—C16X—C17X—C18X	6.0 (19)
C16A—C17A—C18A—C19A	1.3 (9)	C16X—C17X—C18X—C19X	−8(3)
C17A—C18A—C19A—C20A	−1.3 (8)	C17X—C18X—C19X—C20X	5(4)
C18A—C19A—C20A—O1A	−179.6 (4)	C18X—C19X—C20X—O1X	−178 (2)
C18A—C19A—C20A—C15A	1.5 (6)	C18X—C19X—C20X—C15X	2(3)
C16A—C15A—C20A—O1A	179.3 (3)	C16X—C15X—C20X—O1X	176.0 (17)
C14A—C15A—C20A—O1A	−1.5 (5)	C14X—C15X—C20X—O1X	−2(3)
C16A—C15A—C20A—C19A	−1.7 (5)	C16X—C15X—C20X—C19X	−5(3)
C14A—C15A—C20A—C19A	177.6 (3)	C14X—C15X—C20X—C19X	177 (2)
C7B—N1B—C1B—C2B	179.5 (2)	C7Y—N1Y—C1Y—C2Y	−179.7 (5)
C7B—N1B—C1B—C6B	0.5 (2)	C7Y—N1Y—C1Y—C6Y	−0.3 (5)
C6B—C1B—C2B—C3B	0.3 (4)	N1Y—C1Y—C2Y—C3Y	178.9 (7)
N1B—C1B—C2B—C3B	−178.6 (3)	C6Y—C1Y—C2Y—C3Y	−0.5 (9)
C1B—C2B—C3B—C4B	−1.0 (6)	C1Y—C2Y—C3Y—C4Y	2.8 (15)
C2B—C3B—C4B—C5B	1.1 (6)	C2Y—C3Y—C4Y—C5Y	−3.9 (16)
C3B—C4B—C5B—C6B	−0.4 (4)	C3Y—C4Y—C5Y—C6Y	2.3 (10)
C4B—C5B—C6B—N2B	178.5 (2)	C7Y—N2Y—C6Y—C5Y	−179.2 (5)
C4B—C5B—C6B—C1B	−0.3 (3)	C14Y—N2Y—C6Y—C5Y	−12.7 (11)
C7B—N2B—C6B—C5B	179.9 (2)	C7Y—N2Y—C6Y—C1Y	0.9 (5)
C14B—N2B—C6B—C5B	14.1 (3)	C14Y—N2Y—C6Y—C1Y	167.4 (9)
C7B—N2B—C6B—C1B	−1.15 (19)	C4Y—C5Y—C6Y—N2Y	180.0 (5)
C14B—N2B—C6B—C1B	−166.9 (2)	C4Y—C5Y—C6Y—C1Y	−0.1 (7)
C2B—C1B—C6B—C5B	0.3 (3)	N1Y—C1Y—C6Y—N2Y	−0.4 (5)
N1B—C1B—C6B—C5B	179.45 (18)	C2Y—C1Y—C6Y—N2Y	179.1 (4)
C2B—C1B—C6B—N2B	−178.71 (17)	N1Y—C1Y—C6Y—C5Y	179.7 (4)
N1B—C1B—C6B—N2B	0.4 (2)	C2Y—C1Y—C6Y—C5Y	−0.8 (7)
C1B—N1B—C7B—N2B	−1.3 (2)	C1Y—N1Y—C7Y—N2Y	0.9 (5)
C1B—N1B—C7B—C8B	−179.37 (17)	C1Y—N1Y—C7Y—C8Y	−179.6 (5)
C6B—N2B—C7B—N1B	1.6 (2)	C6Y—N2Y—C7Y—N1Y	−1.1 (5)
C14B—N2B—C7B—N1B	167.8 (2)	C14Y—N2Y—C7Y—N1Y	−168.0 (8)
C6B—N2B—C7B—C8B	179.87 (15)	C6Y—N2Y—C7Y—C8Y	179.2 (4)
C14B—N2B—C7B—C8B	−13.9 (3)	C14Y—N2Y—C7Y—C8Y	12.3 (10)
N1B—C7B—C8B—C9B	171.53 (17)	N1Y—C7Y—C8Y—C13Y	4.7 (8)
N2B—C7B—C8B—C9B	−6.4 (2)	N2Y—C7Y—C8Y—C13Y	−175.8 (5)
N1B—C7B—C8B—C13B	−4.5 (3)	N1Y—C7Y—C8Y—C9Y	−172.9 (5)
N2B—C7B—C8B—C13B	177.58 (17)	N2Y—C7Y—C8Y—C9Y	6.6 (7)
C14B—N3B—C9B—C10B	−156.83 (19)	C14Y—N3Y—C9Y—C8Y	−26.8 (10)
C14B—N3B—C9B—C8B	27.5 (3)	C14Y—N3Y—C9Y—C10Y	158.2 (8)
C13B—C8B—C9B—N3B	175.78 (17)	C13Y—C8Y—C9Y—N3Y	−176.8 (5)
C7B—C8B—C9B—N3B	−0.3 (2)	C7Y—C8Y—C9Y—N3Y	0.8 (7)
C13B—C8B—C9B—C10B	0.0 (3)	C13Y—C8Y—C9Y—C10Y	−1.7 (8)
C7B—C8B—C9B—C10B	−176.08 (16)	C7Y—C8Y—C9Y—C10Y	175.9 (5)
N3B—C9B—C10B—C11B	−175.40 (18)	N3Y—C9Y—C10Y—C11Y	176.2 (7)
C8B—C9B—C10B—C11B	0.3 (3)	C8Y—C9Y—C10Y—C11Y	1.0 (10)
C9B—C10B—C11B—C12B	−0.7 (3)	C9Y—C10Y—C11Y—C12Y	2.2 (19)
C10B—C11B—C12B—C13B	0.7 (4)	C10Y—C11Y—C12Y—C13Y	−5(3)
C11B—C12B—C13B—C8B	−0.4 (4)	C9Y—C8Y—C13Y—C12Y	−0.8 (15)
C9B—C8B—C13B—C12B	0.0 (3)	C7Y—C8Y—C13Y—C12Y	−178.2 (13)
C7B—C8B—C13B—C12B	175.9 (2)	C11Y—C12Y—C13Y—C8Y	4(3)
C7B—N2B—C14B—N3B	35.6 (3)	C6Y—N2Y—C14Y—N3Y	161.8 (7)
C6B—N2B—C14B—N3B	−160.96 (18)	C7Y—N2Y—C14Y—N3Y	−33.5 (14)
C7B—N2B—C14B—C15B	−86.7 (4)	C6Y—N2Y—C14Y—C15Y	−72.4 (17)
C6B—N2B—C14B—C15B	76.7 (4)	C7Y—N2Y—C14Y—C15Y	92.3 (14)
C9B—N3B—C14B—N2B	−42.1 (3)	C9Y—N3Y—C14Y—N2Y	40.4 (14)
C9B—N3B—C14B—C15B	79.4 (3)	C9Y—N3Y—C14Y—C15Y	−83.9 (11)
N2B—C14B—C15B—C16B	6.9 (6)	N2Y—C14Y—C15Y—C16Y	−41 (2)
N3B—C14B—C15B—C16B	−111.1 (4)	N3Y—C14Y—C15Y—C16Y	80.5 (18)
N2B—C14B—C15B—C20B	−175.8 (4)	N2Y—C14Y—C15Y—C20Y	145.4 (16)
N3B—C14B—C15B—C20B	66.1 (5)	N3Y—C14Y—C15Y—C20Y	−93.4 (19)
C20B—C15B—C16B—C17B	1.1 (5)	C20Y—C15Y—C16Y—C17Y	−1.0 (18)
C14B—C15B—C16B—C17B	178.4 (4)	C14Y—C15Y—C16Y—C17Y	−175 (2)
C15B—C16B—C17B—C18B	0.4 (4)	C15Y—C16Y—C17Y—C18Y	−4.0 (17)
C16B—C17B—C18B—C19B	−1.1 (7)	C16Y—C17Y—C18Y—C19Y	6(3)
C17B—C18B—C19B—C20B	0.3 (9)	C17Y—C18Y—C19Y—C20Y	−4(4)
C18B—C19B—C20B—O1B	176.0 (6)	C18Y—C19Y—C20Y—O1Y	−167 (2)
C18B—C19B—C20B—C15B	1.3 (10)	C18Y—C19Y—C20Y—C15Y	−2(3)
C16B—C15B—C20B—O1B	−176.8 (4)	C16Y—C15Y—C20Y—O1Y	170.4 (15)
C14B—C15B—C20B—O1B	5.8 (7)	C14Y—C15Y—C20Y—O1Y	−16 (2)
C16B—C15B—C20B—C19B	−1.9 (8)	C16Y—C15Y—C20Y—C19Y	4(3)
C14B—C15B—C20B—C19B	−179.3 (6)	C14Y—C15Y—C20Y—C19Y	177.9 (19)

### Hydrogen-bond geometry (Å, °)

**Table:**

*D*—H···*A*	*D*—H	H···*A*	*D*···*A*	*D*—H···*A*
O1A—H1OA···N1B^i^	0.98	1.69	2.641 (3)	162
N3A—H3AB···O1A^ii^	0.86	2.50	3.241 (3)	144
O1B—H1OB···N1A^iii^	0.97	1.79	2.686 (4)	151
N3B—H3BB···O1B^iv^	0.86	2.15	2.974 (4)	160

Symmetry codes: (i) −*x*+1, −*y*+1, −*z*+1; (ii) −*x*+1, −*y*+1, −*z*+2; (iii) −*x*+2, −*y*+1, −*z*+1; (iv) −*x*+2, −*y*+1, −*z*.
